# Outcome of Comparison between Partial Thickness Skin Graft Harvesting from Scalp and Lower Limb for Scalp Defect: A Clinical Trial Study

**DOI:** 10.29252/wjps.10.2.25

**Published:** 2021-05

**Authors:** Mahdi Eskandarlou, Mehrdad Taghipour

**Affiliations:** 1 Department of General Surgery, Faculty of Medicine, Hamadan University of Medical Science, Hamadan, Iran.

**Keywords:** Donor site, Graft, Scalp, Partial thickness

## Abstract

**BACKGROUND:**

Partial-thickness skin graft is the cornerstone for scalp defect repair. Given the potential side effects following harvesting from these sites, this study aimed to compare the outcomes of graft harvesting from scalp and lower limb.

**METHODS:**

This clinical trial was conducted among a sample number of 40 partial thickness graft candidates (20 case and 20 control group) with scalp defect presenting to Plastic Surgery Clinic at Besat Hospital, Hamadan, Iran during 2018-2019. Sampling was done by simple randomization using random digit table. The donor site in case group and control group was scalp and lower limb respectively.

**RESULTS:**

Overall, 28 patients (70%) were male and 12 (30%) were female. Basal cell carcinoma (BCC) and trauma were the most common etiology for the defects. There was a statistically meaningful relationship between two groups regarding the etiology of defect (*P*=0.02). The mean diameter of defect was 24.28±45.37 mm for all of the patients. The difference between diameters of defect in both groups were statistically meaningful while no such difference between graft diameters was seen. The graft “Take” was completely successful in both groups according to evaluations. The level of postoperative pain was lower in the case group compared to the control according to VAS scale and the satisfaction was higher in them per Likert scale.

**CONCLUSION:**

Scalp can safely be used as donor site for skin graft to be used for scalp defects associated with better results and lower complication rates compared to other donor sites.

## INTRODUCTION

Due to its anatomic location scalp is vulnerable to a variety of traumatic events and diseases. Trauma, burn, skin tumors can cause ulcers and defects which require skin grafts, flaps or a combination of two as a method of repair^1^^-^^3^. 

Scalp area is relatively large, on the other hand given its heavy thickness and good blood supply, scalp possesses strong regeneration (re-epithelialization) capability rendering it a suitable option as a donor site for skin graft^4^^, ^^5^. Good epidermal repair potential of scalp, its immobile station and scar coverage by hair growth (camouflage effect) are among the unique features of scalp that make it an appealing donor for skin grafts. Moreover, switching the another surgical field is unnecessary when scalp is used as donor for an adjacent defect which in turn decreases the operation and anesthesia time and confines surgery related complications to one anatomic location^6^. Scar resulting from graft harvesting will be covered by hair, even in case of alopecia the resultant scar is almost identical to patient’s atrophic scalp which seems more acceptable to patient. 

Based on the aforementioned points outcome evaluation of partial thickness skin graft from the scalp for scalp defect was being evaluated in the present study.

## MATERIAL AND METHODS

**Trial design**

This study was a randomized, control clinical trial with two groups. Patients were candidate for scalp repair plastic surgery and were randomly distributed between two groups of case and control (20 patients in each group) by random digits table regardless of age and gender.

**Ethics Approval**

This trial was approved by the Hamadan University of Medical Sciences Ethics Committee (approval no: 224.1397.rec.umsha.ir). Patients were provided with explanation about each method [conventional (harvesting from lower limb) and study (harvesting from scalp)] and advantages and disadvantages of both methods by physician and written informed consent was acquired before entering the study.

**Study setting**

The study performed on 40 patients with scalp defect at Besat Hospital, Hamadan University of Medical Sciences, Hamadan, Iran during Jan 2018 and Dec 2019. (IRCT number20170114031928N2). 

**Eligibility Criteria**

Inclusion criteria were any patient (regardless of age and gender) with scalp defect requiring skin graft repair for primary defects or defects resulting from flap transfer. Exclusion criteria were patient’s refusal, any skin disease precluding graft harvesting (eczema, folliculitis, tumoral or precancerous lesions), scalp scar due to radiation, atrophy or burn. Twenty patients treated with conventional method were designated as control group.

**Intervention**

In cases with suitable wound bed and diameter of ≥ 2cm repair was done directly with partial thickness skin graft, in patients with skull bone exposed however repair with flap from adjacent scalp was needed which the resultant defect was repaired with skin graft. In this study the skin graft was harvested from adjacent scalp. Hair was shaved if needed then the borders of an area of roughly equal size to the defect was marked using methylene blue in parietal, temporal or occipital region, the area then was injected with normal saline to elevate the skin and facilitate harvesting the graft, then the graft was harvested using D80 or D42 automated dermatome or a manual dermatome with an mean thickness of 0.04 inch. The donor site was then covered with a semi occlusive dressing comprising of a layer of petroleum gauze and a layer of sterile gauze. The recipient area was covered with tie over the dressing method according to plastic surgery principles. The head including the donor and recipient sites was then covered with a tubular net bandage as a whole. Almost 50% of scalp area can be used as donor site, so a 50% scalp defect can be repaired using the intact other half. After 24-h donor site dressing except the last layer was removed, after 5 d recipient site dressing was removed and the graft was evaluated.

**Outcomes**

Donor site was evaluated for pain (VAS scale), time to release of petroleum gauze up to postop day 90, time to complete re-epithelialization up to postop day 90 and patient satisfaction with the cosmetic results, scar disappearance in donor site, folliculitis, scab formation and alopecia up to postop day 90 according to protocol. The pain was evaluated using 10 Visual Analog Scale (VAS) in which 0 represents no pain and 10 represents maximum pain. Graft take was evaluated at postop days 5,16,35,90 which was graded as complete take or successful, partial take and no take or failure. Patient satisfaction with graft harvesting from scalp was evaluated with the Likert scale.

**Data analysis**

All gathered data was entered in SPPS version 21 (SPSS Statistics for Windows, ver. 21.0. Armonk, NY: IBM Corp). Data normal distribution was evaluated using Kolmogorov–Smirnov (KS) test first. Then respective statistical tests were used according to variable type (chi-squared for qualitative and t test for quantitative variables). Non-parametric tests were used in case of abnormal data distribution. A *P*-value of <0.05 was deemed meaningful.

## RESULTS

Forty patients participating in this study 28 (70%) were male and 12 (30%) were female with age ranging from 9 to 85. The mean age of all patients was 63.62±09.73 years. Medical record of all patients were reviewed and hypertension and diabetes mellitus were found as the most common comorbidities. The demographic and clinical characteristics of patients categorized as case and control group are displayed in Table 1.

**Table 1:** Demographic and clinical characteristics of patients in case and control group

**Variable**		**Case group**	**Control group**	**Total**
**Mean age**		57.85±8.12	69.40±11.35	63.62±9.73
**Gender**	**Male**	13(65%)	15(75%)	28(70%)
	**Female**	7(35%)	5(25%)	12(30%)
**Diabetes mellitus** **History**		3	3	5
**Hypertension history**		7	5	12
**Systemic infection**		0	0	0
**Vasculitis**		0	0	0
**Hepatic derangement**		0	0	0
**Neuropathy**		1	0	1
**Corticosteroid use**		1	0	1
**Anticoagulant use**		2	3	3

 The anticoagulant agent used by the patients in this study was Aspirin. Scalp defects are caused by a variety of causes, cancer (77.5%) and trauma (17.5%) were the most common etiologic causes in this study. The defect etiology in case and control group in this study is demonstrated in Table 2.

**Table 2:** Etiologic mechanism of scalp defect

**Etiology**		**Case group** **N(%)**	**Control group** **N(%)**	**Total**	***P*** -value*
**Trauma**		6 (30)	1(5)	7(17.5)	0.02
**Tumor**	**SCC**	2(10)	3(15)	5(12.5)	
	**BCC**	10(50)	16(80)	26(65)	
**Electrocution**		1(5)	0	1(2.5)	
**Pressure ulcer**		1(5)	0	1(2.5)	

* Chi-squared test used to comparison

Chi-squared test showed that there is a meaningful correlation between case and control group in regard of etiologic mechanism of defect (*P*=0.02). Clinical characteristics of scalp defect including anatomic location, depth, defect size, graft size are demonstrated in Table 3. 

**Table 3:** Clinical characteristic of scalp defect in case and control group

**Defect and graft characteristics**		**Case**	** Control**	***P*** **-value**
**Anatomic location**	**Frontal**	4	5	0.726
	**Temporal**	7	7	
	**Parietal**	5	7	
	**Occipital**	3	1	
	**Other**	1	0	
**Depth of defect**	**Subcutaneous**	5	4	0.162
	**Galea**	11	13	
	**Periostea**	4	3	
**Mean defect size** (mm)		56.30±27.39	34.45±14.43	0.004
**Mean graft size **(mm)		80.25±36.21	62.00±24.83	0.072

**Fig. 1:**
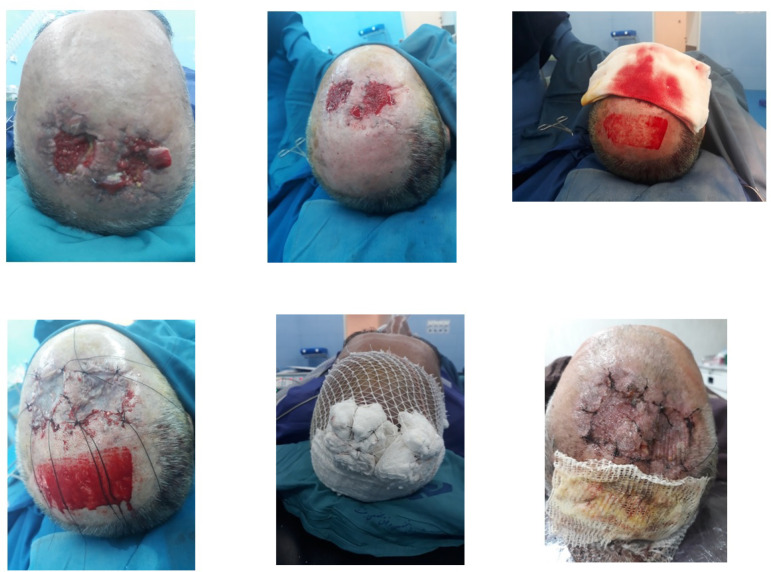
Patient in the case group (scalp donor site) who has undergone a partial thickness skin graft from the adjacent area. Photos were taken during surgery and after treatment

**Figure 2:**
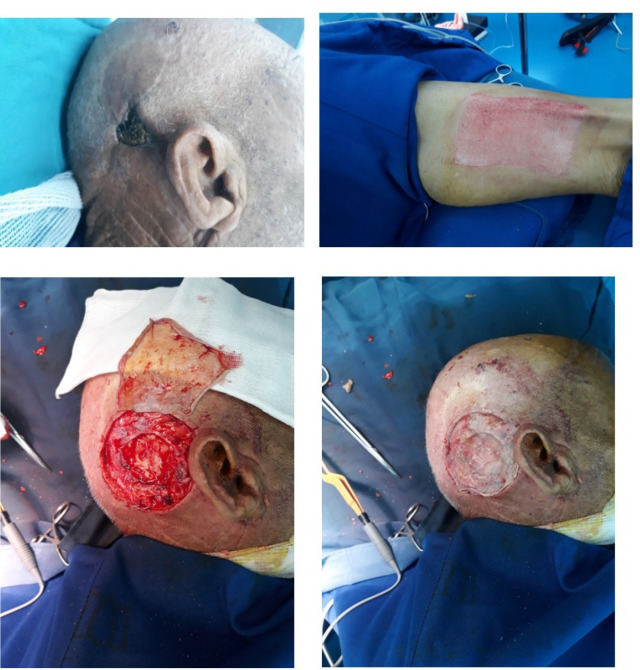
Patient in control group (lower limb donor site)

**Fig. 3:**
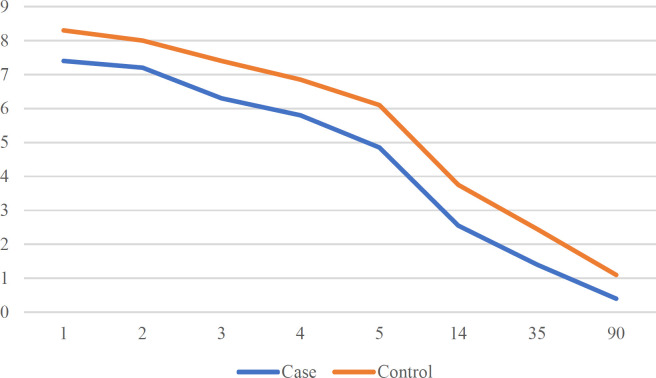
Postoperative pain in case and conrol group

The only statistically meaningful difference in these regard was mean defect size (*P*=0.004). The mean of defect diameter in all patients was 45.37±24.28 mm with minimum diameter of 15 and maximum diameter of 130 mm. The mean diameter of graft in all patients was 71.12±32.1 mm with minimum of 30 and maximum of 170 mm. One of the patients in case group had a 14 mm defect of nasal columella due to BCC repaired with skin graft harvested from scalp. Given the large diameter of defect and exposure of skull in five patients of this study (two in case group both due to cancer and three in control group, two due to cancer and one due to trauma) a combination of flap and graft was used to repair the defect. Harvesting was accomplished from scalp in case group and from lower limb in control group uneventfully. The mean time to release for the petroleum gauze from donor site was 27 d (21 to 23 d) in scalp and 34.2 d (33 to 43 d) in lower limb (*P*=0.00). The donor site was completely healed and epithelialized by the post-op day 90 in both case and control group (Figures 1, 2). In seven patients (35%) of case group crust formation was evident and in one patient (5%) folliculitis or purulent discharge was evident in donor site on day 14 after surgery managed by oral antibiotic and local irrigation. There was no crust formation or folliculitis evident in control group. There was a statistically meaningful difference in postoperative pain level between two groups as was graded using VAS (*P*=0.04). Pain level was evaluated in both group on postop d 1, 2, 3, 4, 5, 14, 35 and 90. The trend of this variable is demonstrated in Figure 3.

Patient satisfaction was evaluated using Likert scale (1 to 5) in which 1 represent dissatisfaction and 5 represent absolute satisfaction. All patients in case group were absolutely satisfied with the donor site scare, of patients in control group 16 were dissatisfied (Likert 1) and four were relatively satisfied, no patient in control group was absolutely satisfied with donor site scar, all these patients were complaining of itching, discoloration and pain and seeking a solution to overcome these complications. Statistical analysis showed a meaningful difference between patient’s satisfaction in two groups (*P*=0.00).

## DISCUSSION

Repair of skin defects associated with tissue loss which are too large to be repaired by primary suturing, is done using skin grafts or flaps. The same rule applies to scalp defects, moreover scalp has some unique features^7^^,^^8^. Thickness of skin and presence of hair follicles in the scalp make it a good donor for partial thickness skin graft. If skull is not exposed the treatment option is skin graft, but in case of skull exposure flap is the answer for this need^9^^-^^11^. Primary repair is dependent on defect diameter, usually scalp defects of smaller than two centimeters are amenable to primary repair but larger defects need skin graft transplant. Skin grafts with partial or full thickness are harvested from lower limb, thigh and neck^12^. Given to its visibility neck is seldom used as a donor for skin graft particularly in children and young adults. The resultant linear scar following a full thickness graft harvest and a superficial scar as discoloration following partial thickness graft harvesting is what patients are always concerned about^13^.

Lower limb is another option the plastic surgeon has in his/her armamentarium but it has its own flaws. Severe pain, discoloration, hypertrophic scar, itching, deformity, pain induced diminished mobility and secondary deep vein thrombosis (DVT) are among the complications of harvesting from lower limb^14^^-^^16^. Donor site discoloration is of great concern, furthermore graft harvested from this donor usually undergoes discoloration and hyperpigmentation in the scalp which is even more concerning in patients with alopecia^17^.

Harvesting skin graft from adjacent scalp for scalp defects will solve most of these problems due to quality and color similarity. Hypertrophic scar formation on the scalp is not common, deformity and itching do not occur and mobility is not impaired but harvesting from scalp^18^. Postoperative pain is also less compared to lower limb donor which will in turn translates in to lower DVT rates. The resultant defect or bald area what make patients skeptical and concerned about harvesting from scalp. Presence of numerous skin appendages and rich blood supply give the scalp the ability of rapid re-epithelialization in superficial layers and collagen and elastin synthesis in deeper layers making donor site repair uneventful and suitable. Presence of hair follicles and hair growth however make scalp prone to crust formation and bacterial contamination^19^^,^^20^. 

We had no harvest induced alopecia in our study as it was predictable. Given the fact that hair follicles are situated in deep layer of scalp not damaged during harvesting partial thickness graft. Crust formation is due to accumulation of exudative fluid and blood clots on top of the donor site^21^. Crust formation occurred in seven patients in case group resolved 35 days after release of the last layer of dressing. The mean time to release of dressing last layer from donor site was lower in case group which was statistically meaningful. This is brought about by rapid re-epithelialization in the scalp. Crust presence in association with the last dressing layer will cause dryness, pain and range of motion limitation, this was particularly prominent in lower limb in control group. The release of the last layer of dressing would diminish all these complications as was the case in case group in this study. Long term severe pain in lower limb donor site following partial thickness skin graft harvesting is essential in patients. The resultant defect from partial thickness graft harvesting acts like a second degree burn which is extremely painful aggravated by adhesive dressing compounded by dried secretion. This may secondarily lead to prolonged limping and limitation in daily activities^22^^-^^24^. Almost every patient complains about donor site complications in the follow up sessions while they are relatively content with the recipient site, almost all of these complications were resolve in our study by harvesting from scalp. We had no complaint about itching or discoloration in donor site after 90 d postoperatively, we also did not have any hypertrophic scar in case group, while most patients in control group were complaining of these complications requiring further measures. 

Absolute satisfaction was documented in all patients in case group from donor site while no patient in control group expressed absolute satisfaction, four patients were relatively satisfied and 16 patients were dissatisfied. Lower pain level, absence of daily activity impairment and acceptable scar color were the reasons for satisfaction in patients in case group. We had one patient with folliculitis due to bacterial contamination in donor site resolved by local irrigation and oral antibiotic therapy. 

## CONCLUSION

The large area, heavy thickness, rich blood supply and numerous skin appendages are the factors tuning the scalp in to a good candidate site for harvesting partial thickness skin graft. The advantages of using scalp as donor are rapid re-epithelialization, no interference with daily activity, less pain, more acceptable scar color and coverage of donor site with hair growth making scalp a suitable option for partial thickness graft for the defects in head and neck.
